# Gut Microbiota Associated With Effectiveness And Responsiveness to Mindfulness-Based Cognitive Therapy in Improving Trait Anxiety

**DOI:** 10.3389/fcimb.2022.719829

**Published:** 2022-02-24

**Authors:** Zonghua Wang, Shuang Liu, Xiaoxiao Xu, Yufeng Xiao, Min Yang, Xiaoyan Zhao, Cancan Jin, Feng Hu, Shiming Yang, Bo Tang, Caiping Song, Tao Wang

**Affiliations:** ^1^ Department of Gastroenterology, Xinqiao Hospital, Army Medical University, Chongqing, China; ^2^ Department of Clinical Nursing, School of Nursing, Army Medical University, Chongqing, China; ^3^ Department of Basic Psychology, School of Psychology, Army Medical University, Chongqing, China; ^4^ Department of Nursing, Xinqiao Hospital, Army Medical University, Chongqing, China

**Keywords:** trait anxiety, gut microbiome, mindfulness-based cognitive therapy, effectiveness, responsiveness

## Abstract

**Objective:**

Mindfulness-based interventions have been widely demonstrated to be effective in reducing stress, alleviating mood disorders, and improving quality of life; however, the underlying mechanisms remained to be fully understood. Along with the advanced research in the microbiota-gut-brain axis, this study aimed to explore the impact of gut microbiota on the effectiveness and responsiveness to mindfulness-based cognitive therapy (MBCT) among high trait anxiety populations.

**Design:**

A standard MBCT was performed among 21 young adults with high trait anxiety. A total of 29 healthy controls were matched for age and sex. The differences in gut microbiota between the two groups were compared. The changes in fecal microbiota and psychological indicators were also investigated before and after the intervention.

**Results:**

Compared with healthy controls, we found markedly decreased bacterial diversity and distinctive clusters among high trait anxiety populations, with significant overgrowth of bacteria such as *Streptococcus, Blautia*, and *Romboutsia*, and a decrease in genera such as *Faecalibacterium, Coprococcus_3*, and *Lachnoclostridium.* Moreover, MBCT attenuated trait anxiety and depression, improved mindfulness and resilience, and increased the similarity of gut microbiota to that of healthy controls. Notably, a high presence of intestinal *Subdoligranulum* pre-MBCT was associated with increased responsiveness to MBCT. Decreases in *Subdoligranulum* post-MBCT were indicative of ameliorated trait anxiety. The tryptophan metabolism pathways were significantly over-represented among high responders compared to low responders.

**Conclusion:**

The significantly increased diversity post-MBCT added evidence to gut-brain communication and highlighted the utility of mycobiota-focused strategies for promoting the effectiveness and responsiveness of the MBCT to improve trait anxiety.

**Clinical Trial Registration:**

chictr.org.cn, ChiCTR1900028389.

## 1 Introduction

As proposed by Spielberger (Spielberger, 1971; Spielberger et al., 2005), trait anxiety has been described as a relatively stable disposition referring to a tendency within individuals to judge a situation as more threatening than it actually is. It has been marked that people with trait anxiety might have excessively anxious and nervous reactions while experiencing dangerous or uncertain situations. Trait anxiety has been reported to be significantly associated with panic attacks, impaired cognitive functions, harm avoidance, obsessive-compulsive disorder, and other psychopathological disorders (Jaiswal et al., 2018; Wang et al., 2018; Hampton, 2019) that eventually result in a reduced quality of life.

According to the diathesis-stress model (Morley, 1983), individuals with elevated trait anxiety inherit a genetic tendency toward a mental disorder. For a more vulnerable person with high trait anxiety, even low environmental stress can trigger a mental illness. In recent years, mindfulness-based interventions have gained worldwide popularity because they have been widely demonstrated to be effective in reducing stress (Fjorback et al., 2011; Khoury et al., 2015), alleviating mood disorders such as depression, anxiety (Ma and Teasdale, 2004; Godfrin and van Heeringen, 2010), and bipolar disorders (Perich et al., 2013), relieving pain and improving the quality of life in clinical or non-clinical populations (Bohlmeijer et al., 2010; Chiesa and Serretti, 2011). Additionally, research has also revealed that mindfulness is negatively associated with trait anxiety, and mindfulness training can improve the latter (Gupta et al., 2006; Sedlmeier et al., 2012; Orme-Johnson and Barnes, 2014). Long-term mindfulness training effectively changes the cortex thickness and the gray matter density in the brain regions, which are associated with learning, memory, attention processes, and mood regulations (Wang et al., 2018; Young et al., 2018).

Despite the growing evidence about the usefulness of mindfulness, the underlying mechanisms are incompletely understood. It has been increasingly suggested that there could be some overlap among the biological, behavioral, and psychosocial mechanisms. Interestingly, the accumulating data have indicated that the gastrointestinal (GI) microbiota can influence brain functions, mood symptoms, and behaviors (Vendrik et al., 2020). The gut microbiota dysbiosis has been observed in individuals with mood disorders, such as autism, bipolar disorder (Gondalia et al., 2019), anxiety, and depression. Therefore, it may be a novel way to explore the gut-brain axis to assist and/or strengthen the effectiveness of psychopharmacology and psychotherapy, such as mindfulness-based interventions. For example, previous studies have reported significant differences in the gut microbiota among patients with major depressive disorder (MDD), as compared to the healthy controls. Their gut microbiota was characterized by a relative abundance of Firmicutes, Actinobacteria, and Bacteroidetes (Cheung et al., 2019; Bastiaanssen et al., 2020). Additionally, following a fecal microbiota transplant from the participants with MDD, depressive-like behaviors were also transferred to germ-free mice. Another animal study found an enhanced physiological response to stress among the germ-free mice as compared to the pathogen-free ones; moreover, the altered stress response could be partially corrected if the GI microbiota were reconstituted at an early age (Gondalia et al., 2019). Furthermore, the consumption of probiotics was demonstrated to have a positive impact on alleviating stress responses, symptoms of anxiety and depression, and cognitive functions in both clinical (Akkasheh et al., 2016; Pinto-Sanchez et al., 2017; Goh et al., 2019) and animal studies (Liu et al., 2020). Therefore, these findings indicate an intimate connection between psychiatry and the GI microbiota. However, there is insufficient evidence to examine the gut microbiome’s functions in the mindfulness interventions’ effectiveness and responsiveness to treat mental illnesses.

Therefore, this study aimed (1) to examine the effectiveness of a mindfulness-based cognitive therapy (MBCT) intervention in relieving trait anxiety, and the changes in the gut microbiota before and after the intervention; and (2) to investigate the potential bacterial genera that may contribute to the responsiveness to the MBCT.

## 2 Methods

### 2.1 Participants and Study Design

This intervention trial was the second phase of the Strong Mind Project. This study was performed at a medical university in China from July 2019 to July 2020. The first phase was a cross-sectional survey to investigate the mental health status of young adults from a medical school. In this phase, questionnaires were distributed to assess the participants’ trait anxiety, mindfulness, depression, resilience, and happiness. Those who scored ≥50 points on the trait anxiety subscale of the State-Trait Anxiety Inventory (STAI) were potentially eligible for the MBCT intervention’s second phase. Eventually, 76 participants in the first phase were invited, of which 41 agreed to complete a further screening to confirm their eligibility for the second phase. The screening rules included the inclusion and exclusion criteria. Those young adults who met the following standards were included: (1) age ≥ 18 years; (2) trait anxiety subscale score ≥ 50 points; (3) willingness and motivation to follow the study protocol; (4) currently not taking an anti-anxiety or anti-depressant medication; and (5) no previous meditation or mindfulness experience. Furthermore, the exclusion criteria were as follows: (1) a history of gastrectomy; (2) a diagnosis of a mental health disorder such as depression and anxiety; (3) the use of anti-acids and/or gastric mucosal protective drugs and/or antibiotics and/or probiotics 4 weeks prior to the study; and (4) inability to follow the MBCT intervention due to physical restraints or other reasons. Additionally, the following were the reasons for a participant to discontinue the study: (1) withdrawal of informed consent; (2) incomplete attendance at the intervention visits due to time conflicts or other reasons; (3) exclusion criteria found after enrollment; and (4) occurrence of any serious adverse events during the intervention period. Accordingly, 25 young adults (intervention group, also known as the high trait anxiety group) were eventually recruited for the intervention, of which 4 participants withdrew from the study during the intervention.

The healthy controls included those young adults with trait anxiety scores lower than 50 points. The inclusion criteria were as follows: (1) age ≥ 18 years; (2) no history of gastrectomy; (3) no current report of depression or anxiety; (4) currently not taking an anti-anxiety or anti-depressant medication; (5) no previous meditation experience; (6) presently not using anti-acids and/or gastric mucosal protective drugs and/or antibiotics and/or probiotics 4 weeks prior to the study; and (7) willing to partake. In total, 29 young adults were included in the study as controls.

### 2.2 Ethical Considerations

The ethical approval was obtained from the medical ethics committee of the Army Medical University (2019-005-02). This trial was registered in the Chinese Clinical Trial Registry (chictr.org.cn, ChiCTR1900028389). The first participant in the intervention study was enrolled on December 30. All participants provided a signed informed consent after the procedures were clearly explained to them.

### 2.3 Mindfulness Intervention

This study performed an MBCT intervention that integrated the elements of mindfulness-based stress reduction into cognitive-behavioral therapy (CBT) (Hofmann and Gómez, 2017). It was originally developed to prevent the relapse of major depressive disorders; however, since then, it has been adapted to diverse populations and contexts. The MBCT differed from other mindfulness interventions in its emphasis on the CBT principles to better detect repetitive negative thinking and to facilitate adaptive cognitive reappraisal. As compared to traditional CBT, it increased the elements of formal mindfulness meditation practices, such as walking meditation and yoga.

Our intervention procedures followed the MBCT’s standard structure, typically taught in a weekly 2- to 2.5-hour group-based format over 8 weeks. The MBCT emphasizes additional participatory exercises and interactive feedback. The eight sessions were discussed in a formal and informal way of troubleshooting and group discussion, including awareness and automatic pilot, living in our own mind, focusing on wandering mind, identification of aversion, acceptance, and letting go, thoughts are not facts, taking care of oneself, and maintaining and extending new learning (Morgan, 2003). Furthermore, daily home meditation practice (approximately 45 min/day) was assigned and expected between sessions. These sessions aimed to build the ability to become increasingly aware of effects, cognitions, and behaviors from an attitude of acceptance and compassion using repetitive attentional training (Lovas and Schuman-Olivier, 2018). The MBCT’s design helped ameliorate cognitive reactivity, such as selective attention to dangerous stimuli and maladaptive rumination, which is prevalent in individuals with high levels of trait anxiety.

### 2.4 Measures

In addition to information on demographics including age, sex, weight, height, body mass index (BMI; weight in kilograms/[height in meters]^2^), 3-day dietary intake, and current use of meditation, self-report questionnaires were also used to assess the participants’ trait anxiety, resilience, mindfulness, and depression.

#### 2.4.1 Trait Anxiety

Personality trait anxiety was described as a relatively stable personality trait with anxious tendencies. In this study, the trait anxiety was measured by the Chinese version of the STAI’s trait anxiety subscale that was originally developed by Spielberger and Gorsuch (Zung, 1974; Goh et al., 2017). It included 20 items rated on a 4-point Likert scale ranging from 1 (almost never) to 4 (almost always). Items 1, 3, 4, 6, 7, 10, 13, 14, 16, and 19 were reverse-scored. The total score for each scale ranged from 20 to 80, with higher scores indicating greater trait anxiety levels.

#### 2.4.2 Resilience

The Connor-Davidson Resilience Scale (Bangsgaard Bendtsen et al., 2012; Dunstan et al., 2017) was employed to assess the participants’ ability to endure stress or pain and to cope with adversity. It comprises 25 items, with each item rated on a 5-point Likert scale ranging from 0 (not at all) to 4 (almost always). The total score ranged from 0 to 100, with higher scores reflecting greater resilience. Furthermore, the scale showed good psychometric properties, with a Cronbach’s *α* value of 0.89 and a test-retest correlation of 0.87.

#### 2.4.3 Mindfulness

The 15-item Mindful Attention Awareness Scale (Duncan et al., 2007) was adopted to measure the participants’ capacity for sustained attention to and awareness of the experience of the present moment in daily life. Its items were reverse-scored and assessed for the absence of mindful attention rather than the actual mindful moments. The scale was scored on a six-point Likert scale, ranging from one (almost always) to six (almost never). A higher score indicated a greater state of mindfulness. The internal consistency of the *α* coefficient was 0.82.

#### 2.4.4 Depression

The 20-item Self-rating Depression Scale (SDS) was used to explore the participants’ self-reported level of depression (Holmstrøm et al., 2004; Jiang et al., 2015). The responses were rated on a four-point Likert scale, ranging from 1 (a little time) to 4 (most of the time), assessing both affective and somatic symptoms. The raw scores that ranged from 20 to 80 were converted to standard scores by dividing the sum of the raw scores by 80 and multiplying the quotient by 100. The higher the overall score, the more frequent the depressive symptoms experienced. Individuals with the SDS standard scores greater than 50 were regarded as having clinically significant anxiety.

### 2.5 Data Collection

The psychometric questionnaires, a 3-day 24-h dietary history, and fresh stool samples were collected from all participants at baseline (before intervention), at week 8 (at the end of the intervention), and at week 12 (4 weeks follow-up of the intervention). The questionnaires were completed through an online website at each time point. The 3-day 24-h dietary history of each participant was recalled and recorded by a trained research assistant. Furthermore, the fecal samples were collected after the questionnaires’ completion. The participants were requested to return the fecal specimen to the research assistant on the day of the sample collection. All stool samples were immediately frozen and stored at −80°C prior to the analysis.

#### 2.5.1 16S Ribosomal Ribonucleic Acid Gene Sequencing

The total genomic deoxyribonucleic acid (DNA) from each fecal sample was extracted using a commercial TIANamp Stool DNA Kit (TIANGEN Biotech Co. Ltd., Beijing, China) based on the manufacturer’s instructions. For sequencing, a DNA fragment comprising the bacterial hypervariable regions V3–V4 of the 16S rRNA gene was amplified with the primers 341F (5’-CCTAYGGGRBGCASCAG-3’, forward primer) and 806R (5’-GGACTACNNGGGTATCTAAT-3’, reverse primer). The PCR amplifications were performed, purified, and subsequently sequenced on an Illumina Miseq high-throughput PE 300 Sequencing platform (Illumina, San Diego, USA) by the Novogene Biomedical Corporation (Beijing, China).

#### 2.5.2 Fecal Microbiota Analysis

The sequence outputs were analyzed using the Quantitative Insights into Microbial Ecology 2 (version 2019. 7) software package on our Linux server (i7-8700K, 64Gb RAM). A bioinformatic analysis process was performed as follows: (1) quality filtering; (2) feature-table rarefaction; and (3) phylogenetic diversity analyses. Subsequently, diversity and composition analyses were carried out for within-sample diversity (α-diversity), between-sample diversity (β-diversity), and principal coordinates analysis (PCoA). Moreover, linear discriminant analysis of effect size (LEfSe) was performed to identify the (a) taxonomic composition of high trait anxiety groups compared to the controls; (b) changes in the taxonomic composition of the gut microbiota in high trait anxiety groups before and after the mindfulness intervention; and (c) taxonomic composition between the “high and low responders” to the mindfulness intervention among the high trait anxiety group. It was considered a significantly discriminant taxa with the LEfSe scores greater than 2.0 (default) and *p* < 0.05. Regarding the “high” and “low” responders to the mindfulness intervention, the former were regarded as those whose score changes on the trait anxiety subscale before and after the intervention were over 50% (*n* = 9); however, the latter were those whose score changes were under 50% (*n* = 11).

#### 2.5.3 Functional Pathway Prediction

The amplicon sequence variants were performed using the Phylogenetic Investigation of Communities by Reconstruction of Unobserved States (version 2) to predict the functional pathways from the 16S rRNA gene sequences. Subsequently, they were categorized into the Clusters of Orthologous Groups and predicted based on the Kyoto Encyclopedia of Genes and Genomes (KEGG) database.

### 2.6 Statistical Analyses

All statistical analyses were conducted using the following software: Statistical Package for the Social Sciences (version 21.0, IBM, US), GraphPad Prism (version 8.0, GraphPad Software Corporate, San Diego, USA), and R (version 3.4.3, Team 2017) with the “ape” and “vegan” packages. The data were presented as means ± standard deviation. With regard to the psychological variables including trait anxiety, mindfulness, resilience, and depression, continuous scores were used in the analyses of the associations with the microbial composition and the correlation analyses with the bacterial abundance. The chi-squared or the Fisher’s exact test was used for assessing the categorical data. Furthermore, the Student’s *t*-test or the analysis of variance was employed for analyzing the continuous data. Significant differences in the α- and β-diversity distances across the groups were examined using the Mann-Whitney *U* test. The *p*-value was set at 0.05 (two-sided) throughout all analyses.

## 3 Results

### 3.1 Sample Characteristics

**Figure 1:**
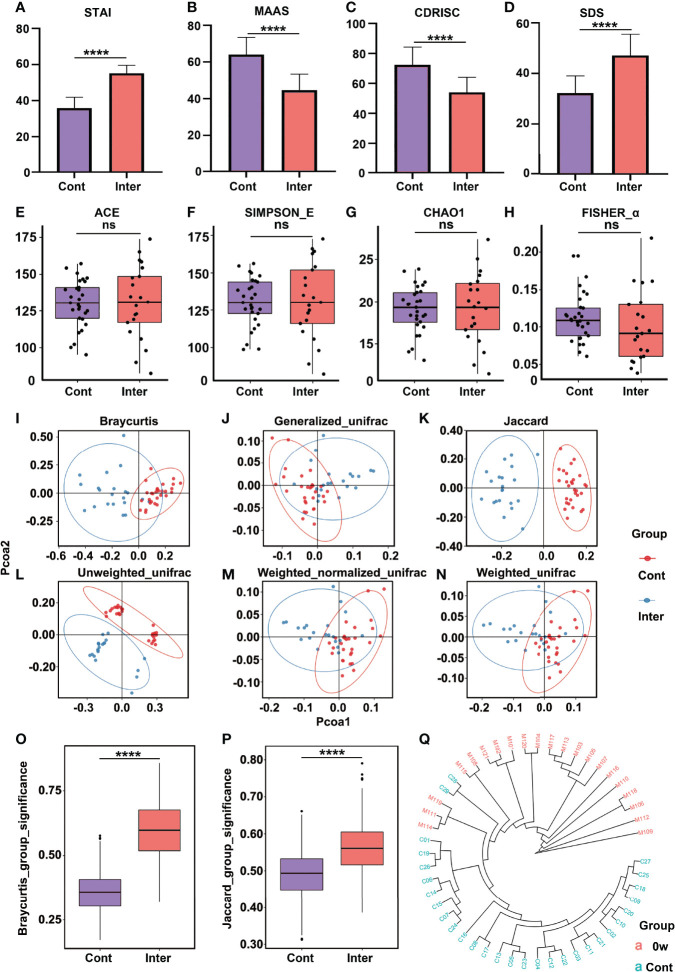
Comparison of psychometric indicators, diversity indices, and PCoA between the intervention group (high trait anxiety) and control group. The mean scores were compared on the scales of STAI **(A)**, MAAS **(B)**, CD-RISC **(C)**, and SDS **(D)**. The α-diversity indices included Ace Index **(E)**, Simpson_E Index **(F)**, Chao1 **(G)**, and Fisher_α **(H)**. The β-diversity indices were illustrated by different algorithms **(I–N)**. Clustering of fecal bacterial communities according to PCoA analysis by different algorithms: the distance indices **(O, P)** and cluster tree **(Q)**. **** means p value < 0.0001; NS means not significant.

**Table 1** presents the demographic characteristics and the descriptive information regarding mental health and daily nutrient consumption at different time points for the high trait anxiety (intervention group) and control groups. The former included 5 women and 16 men, with a mean age of 20.38 years (range: 18-23 years); the latter comprised 7 women and 22 men, with a mean age of 20.97 years (range: 19-25 years). The average BMI was 21.74 and 22.27 for the intervention and control groups, respectively. No significant differences were observed in their age, BMI score, and nutrient intake of protein, fat, carbohydrate, fiber, and sugar at baseline (*p* > 0.05) (**Table 1**). Compared to the healthy controls, the intervention group demonstrated significantly higher levels of trait anxiety (**Figure 1A**) and depression (**Figure 1D**), and lower levels of mindfulness (**Figure 1B**) and resilience (**Figure 1C**) (*p* < 0.001) (**Table 1**).

**Table 1:** Demographics and information on mental health and daily nutrients at each time points.

Variables	MBCT intervention (*n* = 21)	Healthy controls (*n* = 29)	p
	Mean (SD)	Mean (SD)	
**Age**	20.38 ± 1.12	20.97 ± 1.72	0.180
**Weight**	64.04 ± 9.02	66.14 ± 10.02	0.450
**BMI (kg/m^2^)**	21.74 ± 1.71	22.27 ± 2.53	0.410
**Trait anxiety scale**			
0 weeks	54.14 ± 3.95	35.90 ± 5.97	0.000
8 weeks	45.90 ± 8.51		
12 weeks	44.86 ± 9.06		
**Mindfulness scale**			
0 weeks	44.67 ± 8.80	64.03 ± 9.44	0.000
8 weeks	53.48 ± 9.71		
12 weeks	51.95 ± 13.08		
**Resilience scale**			
0 weeks	54.05 ± 10.07	72.45 ± 11.83	0.000
8 weeks	73.13 ± 12.97		
12 weeks	71.86 ± 16.43		
**Depression scale**			
0 weeks	47.10 ± 8.43	32.10 ± 6.87	0.000
8 weeks	37.10 ± 9.27		
12 weeks	41.19 ± 11.09		
**Protein (g)**			
0 weeks	81.40 ± 67.87	78.80 ± 39.31	0.865
8 weeks	51.44 ± 32.73		
12 weeks	76.89 ± 36.01		
**Fat (g)**			
0 weeks	52.65 ± 35.12	54.71 ± 34.27	0.836
8 weeks	34.26 ± 36.49		
12 weeks	56.33 ± 30.90		
**Carbohydrate (g)**			
0 weeks	204.64 ± 123.36	198.26 ± 128.98	0.861
8 weeks	143.96 ± 71.69		
12 weeks	258.10 ± 183.49		
**Fiber (g)**			
0 weeks	6.46 ± 4.23	7.53 ± 6.42	0.509
8 weeks	4.51 ± 4.65		
12 weeks	5.94 ± 4.72		
**Sugar (g)**			
0 weeks	21.20 ± 23.65	12.39 ± 17.41	0.135
8 weeks	13.34 ± 21.19		
12 weeks	19.77 ± 30.78		
**Calories (kcal)**			
0 weeks	1,625.55 ± 963.43	1599.37 ± 869.89	0.920
8 weeks	1,088.46 ± 691.40		
12 weeks	1,848.15 ± 991.19		

MBCT, mindfulness-based cognitive therapy; BMI, body mass index; SD, standard deviation.

### 3.2 Different Gut Microbiota Compositions Prior to the MBCT

#### 3.2.1 Fecal Bacterium Composition

**Figure 2:**
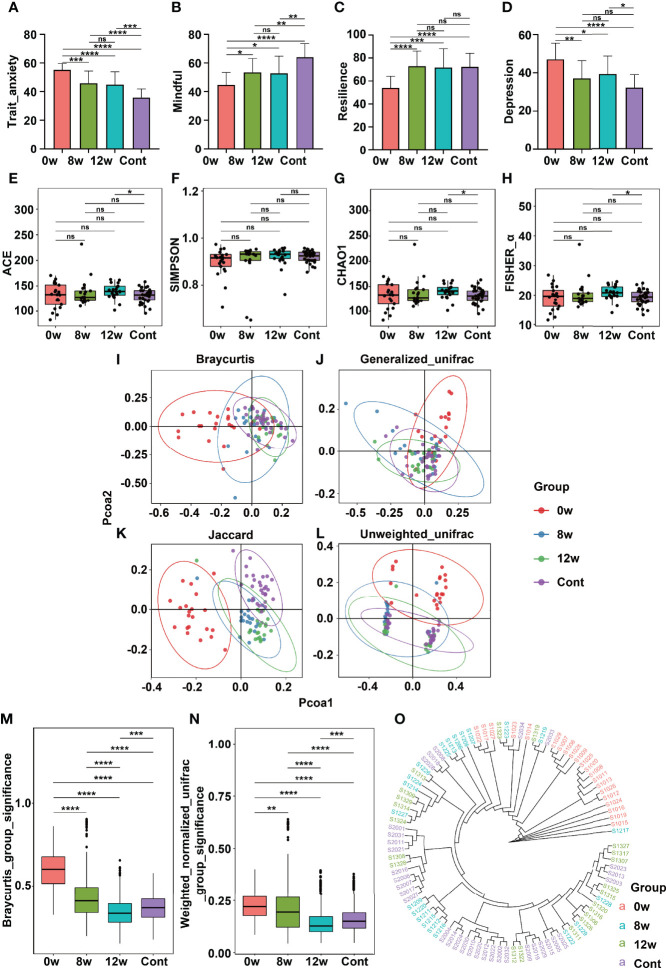
Comparison of psychometric indicators, diversity indices, and PCoA in different time points before and after MBCT intervention (at baseline, at the end of intervention, and at 4 weeks following the end of intervention). The scores were compared on scales of STAI **(A)**, MAAS **(B)**, CD-RISC **(C)**, and SDS **(D)**. The α-diversity indices included Ace Index **(E)**, Simpson_E Index **(F)**, Chao1 **(G)**, and Fisher_α **(H)**. The β-diversity indices were illustrated according to PCoA analysis by different algorithms **(I–L)**. Clustering of fecal bacterial communities: the distance indices **(M, N)** and cluster tree **(O)**. * means p value <0.05; **means p value <0.01; *** means p value <0.001; **** means p value <0.0001; ns means not significant.

There was no significant difference in the α-diversity between the intervention and control groups (**Figures 1E** **–H**). However, significant differences were observed in the β-diversity index, indicating the extent of similarity in the microbial communities. As shown in **Figures 1I–N**, the gut microbiota of the two groups could be divided into two distinct clusters and separated clearly by the PCoA analysis. To further evaluate whether the high levels of trait anxiety had an effect on the β-diversity, we compared the distance between each pair of samples between the groups; significantly greater unweighted unique fraction metric (UniFrac) distance was observed in the healthy controls (*p* < 0.001) (**Figures 1O, P**). The cluster tree of each fecal sample between the two groups are shown in **Figure 1Q**. It is clearly separated into two groups, with red and blue colors representing the features in the high trait anxiety group before intervention and those in healthy controls, respectively. **Supplementary Figure 1** displays the difference in the relative abundance of fecal microbiota between the two groups at the phylum, family, and genus levels. A cladogram showing differences in the fecal microbiota between them is presented in **Supplementary Figures 2A, C**. Furthermore, as shown in **Figure 2C**, the *Actinobacteria* phylum was greatly enriched in the healthy controls, while *the Firmicutes* phylum was consistently higher in the high trait anxiety group.

#### 3.2.2 Gut Microbiota Difference

To further investigate the significant bacteria that may contribute to the differences in the gut microbiota between the high trait anxiety group and the healthy controls, we performed a partial least square discriminant analysis (PLS_DA). The top five bacterial genera that met the standard of vipscore > 2 were *Streptococcus, Blautia, Romboutsia, Lachnoanaerobaculum*, and *Lachnoclostridium* (**Supplementary Figure 3C**). Further comparisons were performed on the relative abundance of all significant bacteria between the high trait anxiety group and the healthy controls (**Supplementary Figure 4**). As compared to the latter, the former showed significantly lower relative abundances of *Streptococcus, Blautia, Romboutsia, Escherichia_Shigella, Eubacterium_hallii_group, Eggerthella*, and *Allorhizobium_Neorhizobium_Pararhizobium_Rhizobium*; furthermore, they indicated significantly higher relative abundances of *Lachnoanaerobaculum*, *Lachnoclostridium, Rothia, Leptotrichia, Lachnospiraceae_UCG_010, Faecalibacterium, Coprococcus_3, Eubacteriumeligens_group, Atopobium, GCA_900066575*, and *Pseudopropionibacterium.*

### 3.3 The MBCT Intervention Altered Gut Microbiota

As compared to the baseline (time point: 0 weeks), the intervention group participants experienced significant reductions in trait anxiety (**Figure 2A**), significant improvements in mindfulness (**Figure 2B**), significant increase in resilience (**Figure 2C**) and significant decrease in depression (**Figure 2D**) at the end of the intervention (time point: 8 weeks) and at the 4-week follow-up (time point: 12 weeks) (all *p* < 0.05). The community richness and diversity of the fecal microbiome shown by the α-diversity indices did not vary significantly before and after the MBCT intervention (**Figures 2E** **–H**). Nevertheless, the intervention group displayed a decreasing β-diversity distance over the time points, indicating that the MBCT intervention had a significant effect on the gut microbiota. In accordance with these observations, the PCoA demonstrated changes in the pattern of clustering over the period of the intervention and the follow-up. Pre- and post-intervention, the gut microbiota was separated into different clusters among the high trait anxiety group (**Figures 2I** **–L**). Interestingly, at the end of the intervention (8 weeks) and at the 4-week follow-up (12 weeks), the β-diversity among this group changed more similarly to that among the healthy controls (**Figures 2M, N**). The cluster tree of each fecal sample is shown in **Figure 2O**. A cladogram indicating differences in the fecal microbiota among the intervention group over different time points is presented in **Supplementary Figure 2**. Notably, at the intervention’s end (week 8), they reported an increase in the abundance of the phyla *Actionbacteria*, *Proteobacteria*, and *Fusobacteria* (**Supplementary Figure 2D**).

#### 3.3.1 Changes of the Significant Genera Over the Intervention Period

The relative abundance of the 18 bacteria was found to be significant between the intervention and control groups over the intervention period (**Supplementary Figure 5**). Specifically, from the baseline to the end of the follow-up, the MBCT intervention resulted in a significant increase in the relative abundances of *Streptococcus, Blautia, Romboutsia*, and *Eggerthella*, but a significant decrease in that of *Lachnoclostridium, Rothia, Lachnospiraceae_UCG_010, Faecalibacterium, Coprococcus_3*, and *Eubacteriumeligens groups*. Additionally, at its end (week 8) and at the 4-week follow-up (week 12), the relative abundances of the abovementioned bacteria were more similar to those in the healthy controls; this suggested that the MBCT intervention could successfully shift the high trait anxiety microbiota community toward that of the healthy controls.

### 3.4 *Subdoligranulum* Relating to the Responsiveness to the MBCT Intervention

**Figure 3:**
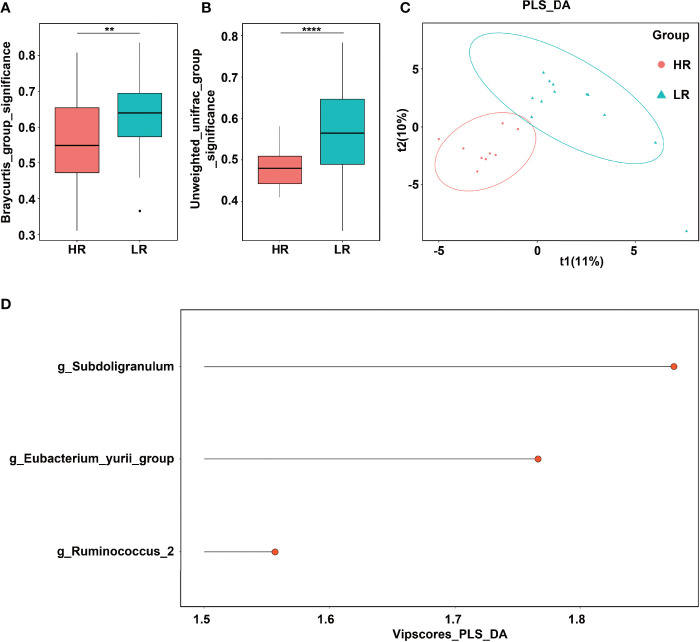
Significant genera that contributed to response strength to mindfulness intervention. **(A)** Bray–Curtis distance. **(B)** Unweighted_unifrac distance. **(C)** PLS_DA analysis. **(D)** Significant genera with vipscore > 2 in PLS_DA analysis. ** means p value < 0.01; **** means p value < 0.0001.

To evaluate whether the responsiveness to the MBCT intervention was related to the gut microbiota, we compared the bacterial profiles between the high- and low-responder groups. There were no significant differences in α-diversity between the low-responder group and high-responder group before MBCT (0 weeks) and after MBCT (8 weeks) (**Supplementary Figure 6**). The Bray–Curtis and Unweighted_unifrac distances suggested significantly lower β-diversity distances in the high-responder group than in the low-responder one (**Figures 3A, B**) (*p* < 0.01). The PCoA plots revealed significant separation based on the genotype (**Figure 3C**). Moreover, **Supplementary Figure 7** displays the difference in the relative abundance of the fecal microbiota between the high and low responders at the phylum, family, and genus levels. Although no significant difference was revealed in the aspect of α-diversity, a significantly different cluster was detected through the PCoA analysis in the high-responder group (**Supplementary Figure 8**).

**Figure 4:**
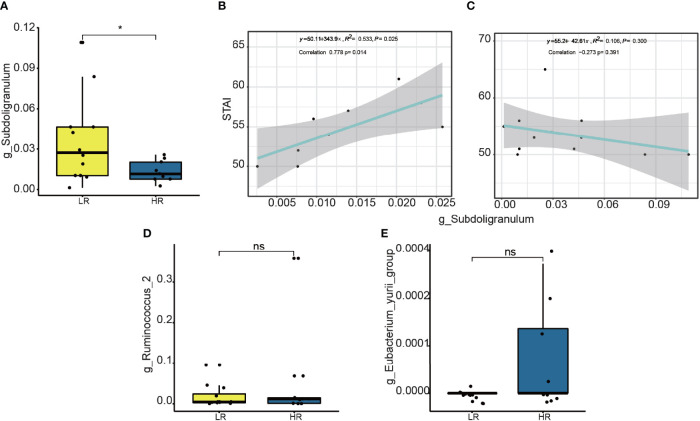
Difference on relative abundance of significant genera between high responders (HR) and low responders (LR): *Subdoligranulum* **(A)**, *Ruminococcus* **(D)**, and *Eubacterium_yurii_group* **(E)**. The linear correlation between the relative abundance of the genera *Subdoligranulum* and STAI scores is shown HR **(B)** and LR **(C)**. * means p value <0.05; ns means not significant.

The significant bacteria with vipscores > 2 in the PLS_DA analysis were *Subdoligranulum, Eubacterium_yurri_group*, and *Ruminococcus* (**Figure 3D**). Furthermore, a comparison was performed on the relative abundance of the abovementioned three bacteria between the high and low responders (**Figures 4A, D, E**). A significantly lower relative abundance of *Subdoligranulum* was reported in the former than in the latter (**Figure 4A**) (*p* < 0.05), whereas no significant difference was found in the other two bacteria (**Figures 4D, E**). This suggested that the higher the level of the *Subdoligranulum* genus, the lower the response strength to mindfulness intervention.

To determine whether the *Subdoligranulum* levels were related to the trait anxiety, we performed linear correlation analyses. As shown in **Figure 4B**, the relative abundance of the operational taxonomic units (OTUs) in the *Subdoligranulum* was positively correlated with the trait anxiety scores in the high responders; this finding suggested that the higher the *Subdoligranulum* level, the higher the STAI score. This signified that a higher *Subdoligranulum* level implied a lower responsiveness to the MBCT intervention, leading to a greater level of trait anxiety. This was also demonstrated by the negative correlation between the *Subdoligranulum* abundance and trait anxiety (STAI score) in the low responders (**Figure 4C**). The linear correlation between the high and low responders in the *Subdoligranulum* levels with other psychometric indicators (mindfulness, resiliency, and depression) is presented in **Supplementary Figure 9**.

**Figure 5:**
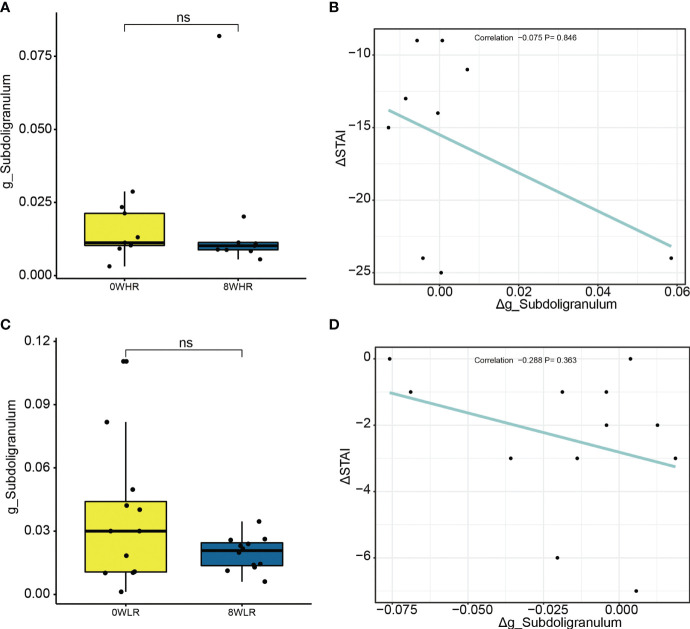
Difference on relative abundance of *Subdoligranulum* genera before and after the intervention among high responders **(A)** and low responders **(C)**, respectively. The linear correlation between Δg_*Subdoligranulum* and ΔSTAI score among high responders **(B)** and low responders **(D)**, respectively. ns means not significant.

To determine the role of *Subdoligranulum* in affecting the responsiveness, a further linear correlation analysis was conducted between the OTUs and the score difference on the trait anxiety before and after the MBCT intervention (week eight minus week zero). Although no significance was observed (**Figure 5A**), a negative correlation was illustrated in the high responders between the *Subdoligranulum* abundance and the difference in the STAI scores (**Figures 5C, D**). No significant difference between the Therefore, the genus *Subdoligranulum* was considered a potential target that may help strengthen the MBCT intervention response.

### 3.5 Tryptophan Metabolism Over-Represented Among the High Responders

**Figure 6:**
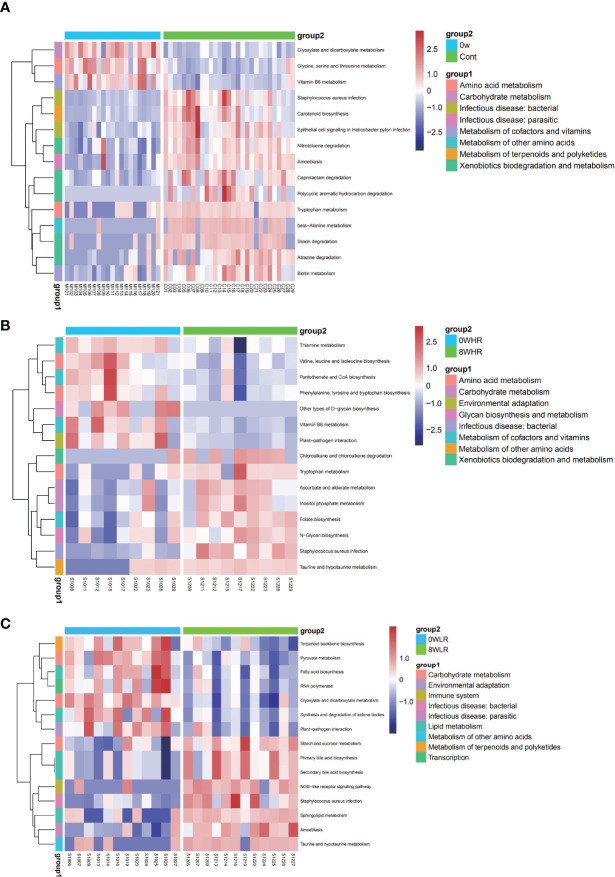
Heat maps of selected significant metabolites between the high trait anxiety group and healthy control group **(A)**, before and after intervention among high responders **(B)** and among low responders **(C)**.

**Figure 7:**
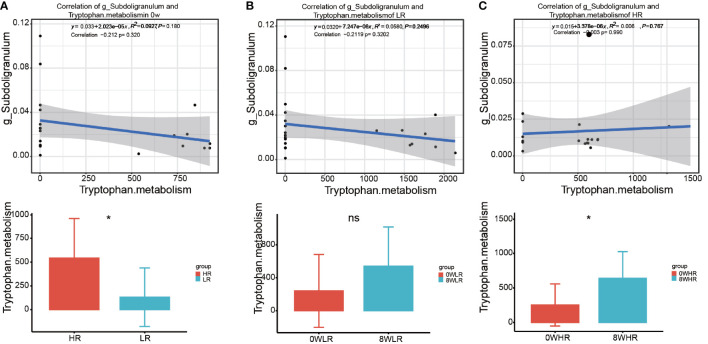
The correlation of the taxa *Subdoligranulum* with Tryptophan metabolism pathway between high responders and low responders **(A)**, in low responders between 0 week and 8 week **(B)**, and in high responders between 0 week and 8 week **(C)**. * means p value <0.05; ns means not significant.

As shown in **Figure 6**, this study adopted the KEGG pathway analysis to detect the relative abundances of the functional genes in the gut microbiota that may affect the response strength. The color values in the heat map represent the normalized relative abundance of the KEGG pathways. The analysis indicated that genes for the tryptophan metabolism were significantly over-represented among the high responders as compared to the low ones (**Figure 6A**). In addition, a further comparison of the KEGG pathways between the two time points at baseline (week zero) and after the MBCT intervention (week eight) were conducted in the high- and low-responder groups, respectively. In the former, a significant enrichment in the *Staphylococcus aureus* infection, taurine and hypotaurine metabolism, ascorbate and aldarate metabolism, tryptophan metabolism, and nitrotoluene degradation were found post-intervention (week 8), as compared to the baseline (**Figure 6B**). In low responders, it was observed that a gene family linked to the *S. aureus* infection, taurine and hypotaurine metabolism, and nitrotoluene degradation were significantly increased post-intervention (week 8), as compared to the baseline (**Figure 6C**); however, there was no obvious difference in the tryptophan metabolism pathway in them. Overall, these data suggested that the gut microbiota from the high responders was significantly enriched with the genes for tryptophan metabolism, which may be a potential pathway affecting the response strength toward the MBCT intervention. Furthermore, we detected a trend of negative correlation between the taxa *Subdoligranulum* with tryptophan metabolism pathway in the low-responder group and a reversed correlation in the high-responder group (**Figure 7**).

## 4 Discussion

Although an increasing number of studies have reported an altered gut microbiome composition among the patients with MDD, its profile among individuals with high trait anxiety was unclear thus far. Thus, this study revealed significant alterations in the abundance of the different genera within the Lachnospiraceae, Peptostreptococcaceae, and Ruminococcaceae families. In particular, compared to the healthy controls, we found a significantly decreased abundance of *Streptococcus*, *Blautia*, *Romboutsia*, and *Eggerthella*, and conversely, a significantly increased abundance of *Lachnoanaerobaculum*, *Lachnoclostridium*, *Faecalibacterium*, and *Coprococcus.* In this research, the elevated expression of the Lachnospiraceae family (belonging to the phylum Firmicutes) was found in people with high trait anxiety. Consistent with previous reports in animal studies, the abundance of the Lachnospiraceae family was correlated with behavioral changes induced by stress (Bangsgaard Bendtsen et al., 2012). This phenomenon may be explained by the mechanism through which the Lachnospiraceae family participates in the breakdown of carbohydrates into the microbiota-derived short-chain fatty acids (SCFAs) (Duncan et al., 2007); therefore, an increase in the Lachnospiraceae family promotes a decrease in the SCFA concentration, which, in turn, causes an intestinal barrier dysfunction (Jiang et al., 2015). It is widely acknowledged that the SCFAs, such as acetate, propionate, and butyrate, can promote an epithelial barrier function in the large intestine (Morris et al., 2017; Bojović et al., 2020).

The *Subdoligranulum* taxa, a Gram-negative, non-motile, and non-spore-forming microbe, belongs to the Clostridia family. As an SCFA-producing taxon, the major products of *Subdoligranulum* are butyric and lactic acids, composed of minor amounts of acetic and succinic acids (Holmstrøm et al., 2004). However, its functions and roles in different diseases remain inconsistent. Moreover, its richness and abundance were found to be positively associated with asthma (Agus et al., 2018) and diabetes mellitus. Interestingly, a significantly enriched relative abundance of *Subdoligranulum* was revealed among participants receiving long-term meditation training, as compared to those who did not receive any meditation training (Jia et al., 2020). The MBCT has emphasized formal mindfulness meditation practices, such as walking meditation and yoga (Lovas and Schuman-Olivier, 2018). This might explain why we found a positive correlation between the *Subdoligranulum* genera and the MBCT’s responsiveness. In contrast, other studies have demonstrated under-expression of the genus *Subdoligranulum* among the *Clostridium difficile* infection (Pérez-Cobas et al., 2014), high cancer-related fatigue (Xiao et al., 2021), and food allergy (Abdel-Gadir et al., 2019). In this research, we found that the abundance of *Subdoligranulum* was positively correlated with the trait anxiety scores in the high-responder group. We assumed that the *Subdoligranulum* genera were auxotrophic for the most of the vitamins and the amino acid tryptophan (Soto-Martin et al., 2020), which is generally recognized as a candidate for protecting individuals against stress disorders.

Tryptophan and the related metabolites have a wide range of physiological functions and are involved in the modulation of mood, anxiety, stress response, and social behaviors (Agus et al., 2018). Recent studies have verified the tryptophan–microbiome–immune system interactions, indicating that the gut microbiota can modulate the immune system through the tryptophan metabolism pathways (Gao et al., 2018). Both clinical and rodent research have revealed that an acute tryptophan depletion dramatically inhibits serotonin synthesis and reduces tryptophan concentrations in the brain, resulting in a decrease in the central serotonin and related metabolites such as the 5-hydroxyindoleacetic acid and serotonin 1A receptor binding (Yano et al., 2015). Furthermore, there is strong evidence implicating that a low serotonin synthesis is associated with a depressed mood and an impaired cognitive function (Lai et al., 2021). Through various preclinical strategies, it has been established that manipulating microbial colonization in the GI tract influences tryptophan availability. The serotonin synthesis occurs peripherally within the gut neurons and the enterochromaffin cells as well as centrally within the neurons of the raphe in the brainstem (Jenkins et al., 2016). The serotonin receptors, which are involved in cognitive processes such as learning and memory, have been detected in the brain regions including the cortex, amygdala, and hippocampus. Similarly, in this study, we found an enriched expression of the tryptophan metabolism pathways among the high-response participants, as compared to those with a low response to the MBCT intervention through the KEGG pathway analysis. This finding suggested that the tryptophan metabolism in the gut microbiota may potentially be a pathway to promote response strength to psychotherapy, such as a mindfulness-based program, in the brain. In future studies, animal models could be developed to investigate the definite mechanisms of the tryptophan pathways in the interaction between the gut microbiota and the MBCT intervention.

This is the first exploratory study to explore the possible functions of the gut microbiota in an MBCT intervention. However, it had certain limitations. First, as this was a pilot research, it intended to assess the feasibility, acceptability, and preliminary efficacy of mindfulness from the perspective of the gut microbiota; moreover, we focused on how the gut microbiota changed before and after the MBCT intervention. Although we set a healthy control group at baseline, we failed to trace the changes in their gut microbiota and did not collect their parallel data after the intervention. This might reduce the validity of the results to prove a definite effect of the MBCT on the gut microbiota. Second, this study utilized a relatively small homogenous sample of young adults that might limit our generalizability to other groups and potentially prevent us from obtaining other statistically significant results. However, considering the small sample size, we were able to find significant results.

## 5 Conclusion

The current study highlighted differences in the gut microbiota between the high trait anxiety individuals and the healthy controls. This was a valuable attempt to fill the research gap regarding finding a significant intestinal microbiome that may affect trait anxiety in young adults. Furthermore, these preliminary results provide information for further intervention studies to reduce mental disorders from the perspective of the gut microbiota. Moreover, we further explored the gut microbiota changes during the effectiveness of the MBCT intervention, thus contributing evidence to the current mechanism of the gut-brain axis of how psychotherapy improves mental health. We also examined the potential bacterial genera and the possible pathways that influence the responsiveness to the MBCT intervention among the high trait anxiety individuals. Notably, the preexisting high abundance in the populations with high trait anxiety prior to the MBCT was associated with increased bacterial diversity and enhanced therapeutic outcomes. These findings support the feasibility of combining gut microbiota therapy with MBCT to strengthen the effectiveness of alleviating trait anxiety in this population.

## Data Availability Statement

The original contributions presented in the study are publicly available in NCBI using accession number PRJNA751268.

## Ethics Statement

Ethical approval was obtained from the Medical Ethics Committee of Army Medical University (2019-005-02). This trial was registered at the Chinese Clinical Trial Registry (Chictr.org.cn, ChiCTR1900028389). The patients/participants provided their written informed consent to participate in this study.

## Author Contributions

ZW, XX, BT, CS, and TW conceived and designed this study. ZW, XX, CJ, FH, CS, and TW performed the intervention and collected data. ZW, SL, YX, MY, and BT collected and analyzed fecal samples. SL and BT carried out statistical analysis. All authors contributed to data interpretation. ZW prepared the manuscript. ZW, XZ, SL, SY, CS, and TW revised the manuscript. All authors contributed to the article and approved the submitted version.

## Funding

Military Medical Science and Technology Foundation, Grant Number: 2019ZLX002; Army Medical Science and Technology Programme, Grant Number: 20QNPY005; Army Medical University Specialized Scientific Research Fund, Grant Number: 2019XYY17; and Joint Research Grant by Chongqing Science Foundation and Health Committee, Grant/Award Number: 2020FYYX035.

## Conflict of Interest

The authors declare that the research was conducted in the absence of any commercial or financial relationships that could be construed as a potential conflict of interest.

## Publisher’s Note

All claims expressed in this article are solely those of the authors and do not necessarily represent those of their affiliated organizations, or those of the publisher, the editors and the reviewers. Any product that may be evaluated in this article, or claim that may be made by its manufacturer, is not guaranteed or endorsed by the publisher.
