# Supplementary material for: Gut Microbiota Associated With Effectiveness And Responsiveness to Mindfulness-Based Cognitive Therapy in Improving Trait Anxiety
Source: Front Cell Infect Microbiol. 2022 Feb 24;12:719829. doi: 10.3389/fcimb.2022.719829 (PMC8908961; doi:10.3389/fcimb.2022.719829)

Supplementary Figure 1. Relative abundances between the high trait-anxiety group (S1006-S1029) and the healthy controls (S2001-S2034) at the phylum level, the family level and the genus level of bacterial composition. (A) Relative abundance at the phylum level. (B) Relative abundance at the family level. (C) Relative abundance at the genus level.


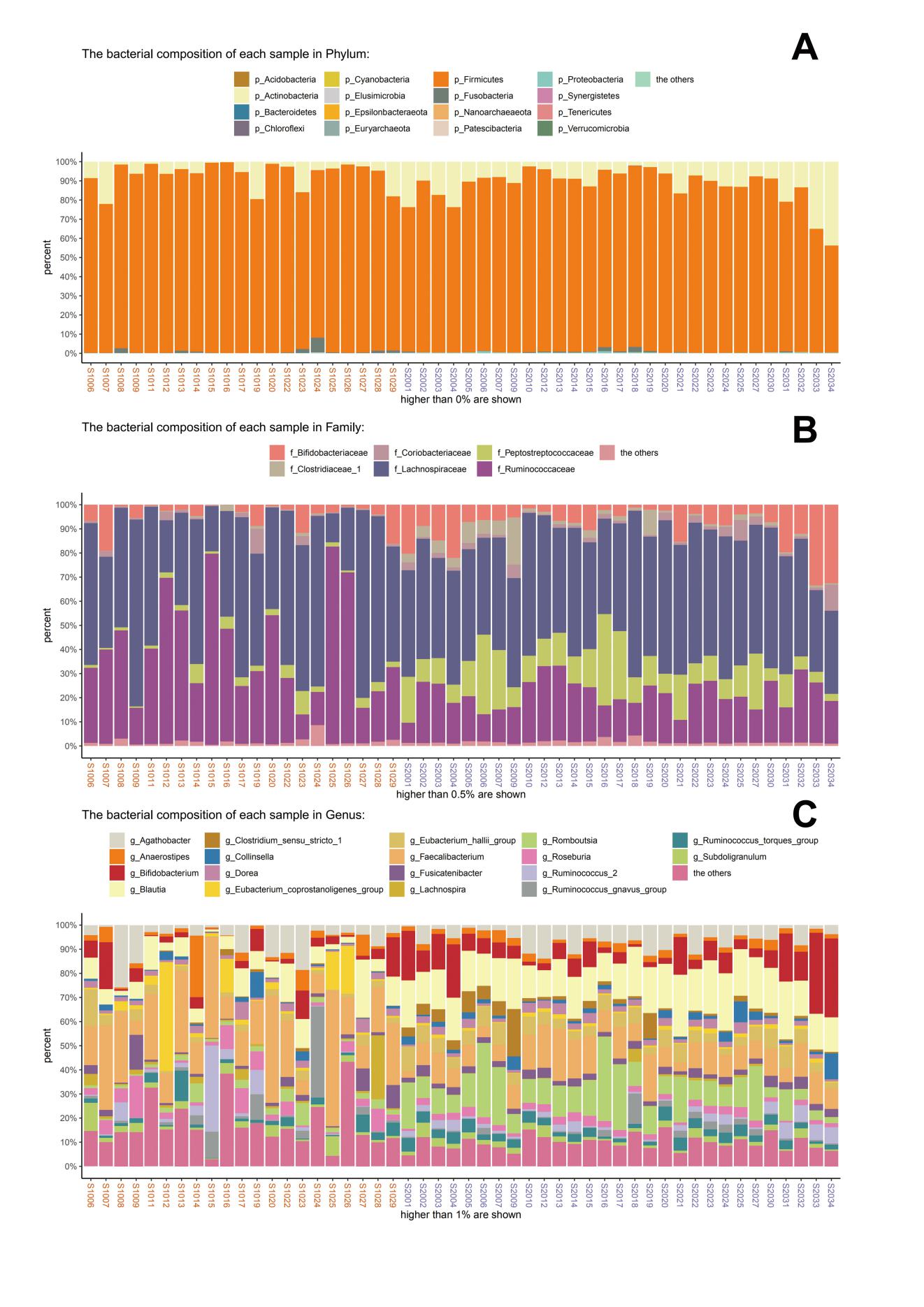


Supplementary Figure 2. Different structures of gut microbiota obtained from LEfSE analysis. A histogram of the LDA scores for the most abundant phylotypes between high trait-anxiety group and healthy control group (A) and among high trait-anxiety group over different time points (C). Only taxa meeting an LDA significant threshold > 2 were shown. Taxonomic cladograms between high trait-anxiety group and healthy control group (B) and among high trait-anxiety group over different time points (D). The diameter of each dot is proportional to its effect size.


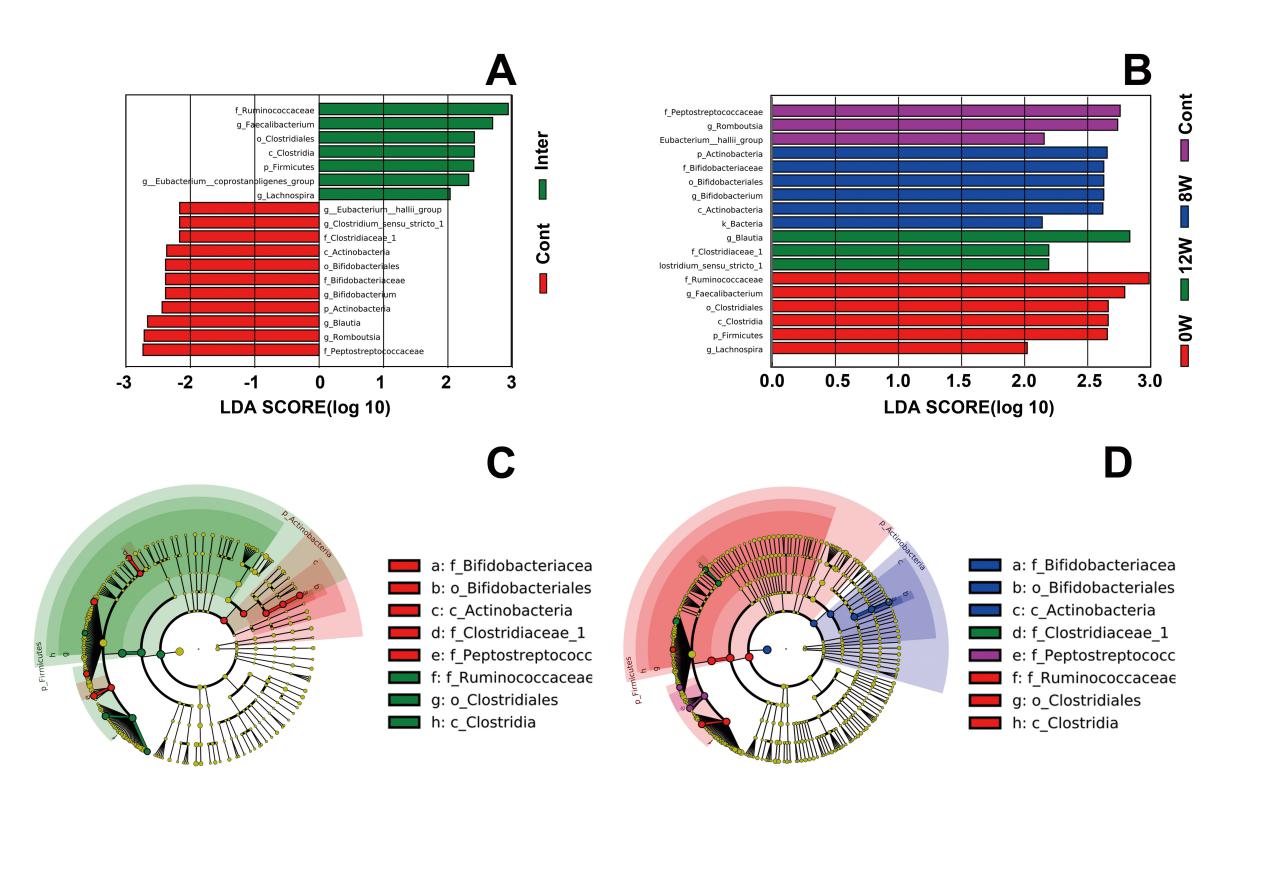


Supplementary Figure 3. Significant bacterial genera that contributed to gut microbiota difference between between high trait-anxiety group and healthy control group. (A) OPLS_DA analysis. (B) Bray-Curtis distance. (C) Significant genera with vipscore ＞ 2 in OPLS_DA analysis.


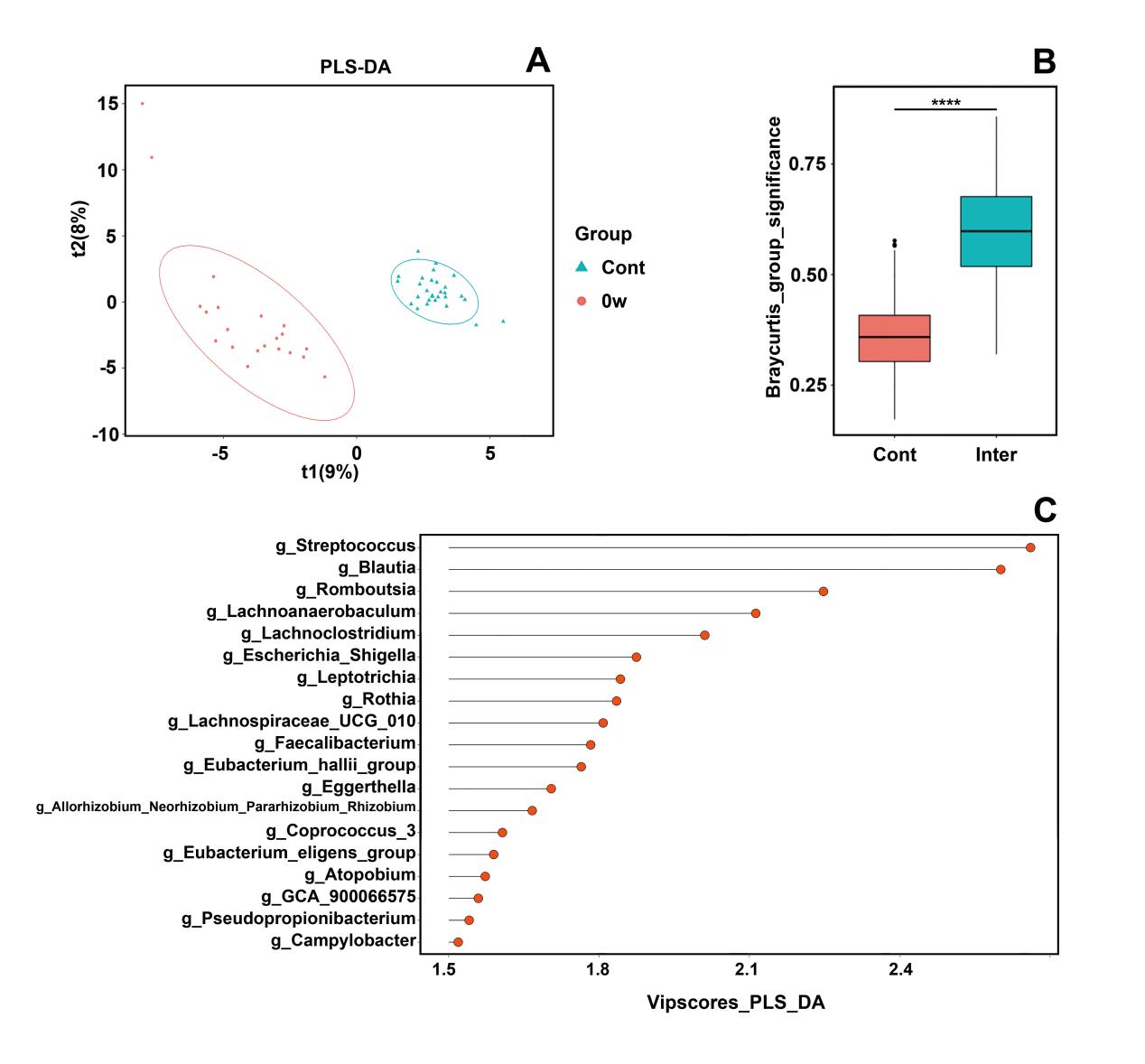


Supplementary Figure 4. Relative abundance of significant genera between high trait-anxiety group and healthy control group. Lower relative abundance among high trait-anxiety group: *Streptococcus* (A)*, Blautia* (B)*, Romboutsia* (C)*, Escherichia_Shigella* (F)*, Eubacterium_hallii_group* (K)*, Eggerthella* (L)*,*and *Allorhizobium_Neorhizobium_Pararhizobium_Rhizobium* (M)*.* Higher relative abundance among high trait-anxiety group: *Lachnoanaerobaculum* (D)*, Lachnoclostridium* (E), *Rothia* (G)*, Leptotrichia* (H)*, Lachnospiraceae_UCG_010* (I)*, Faecalibacterium* (J)*, Coprococcus_3* (N)*, Eubacterium_eligens_group* (O)*, Atopobium* (P)*, GCA_900066575* (Q) and *Pseudopropionibacterium* (R)*.*


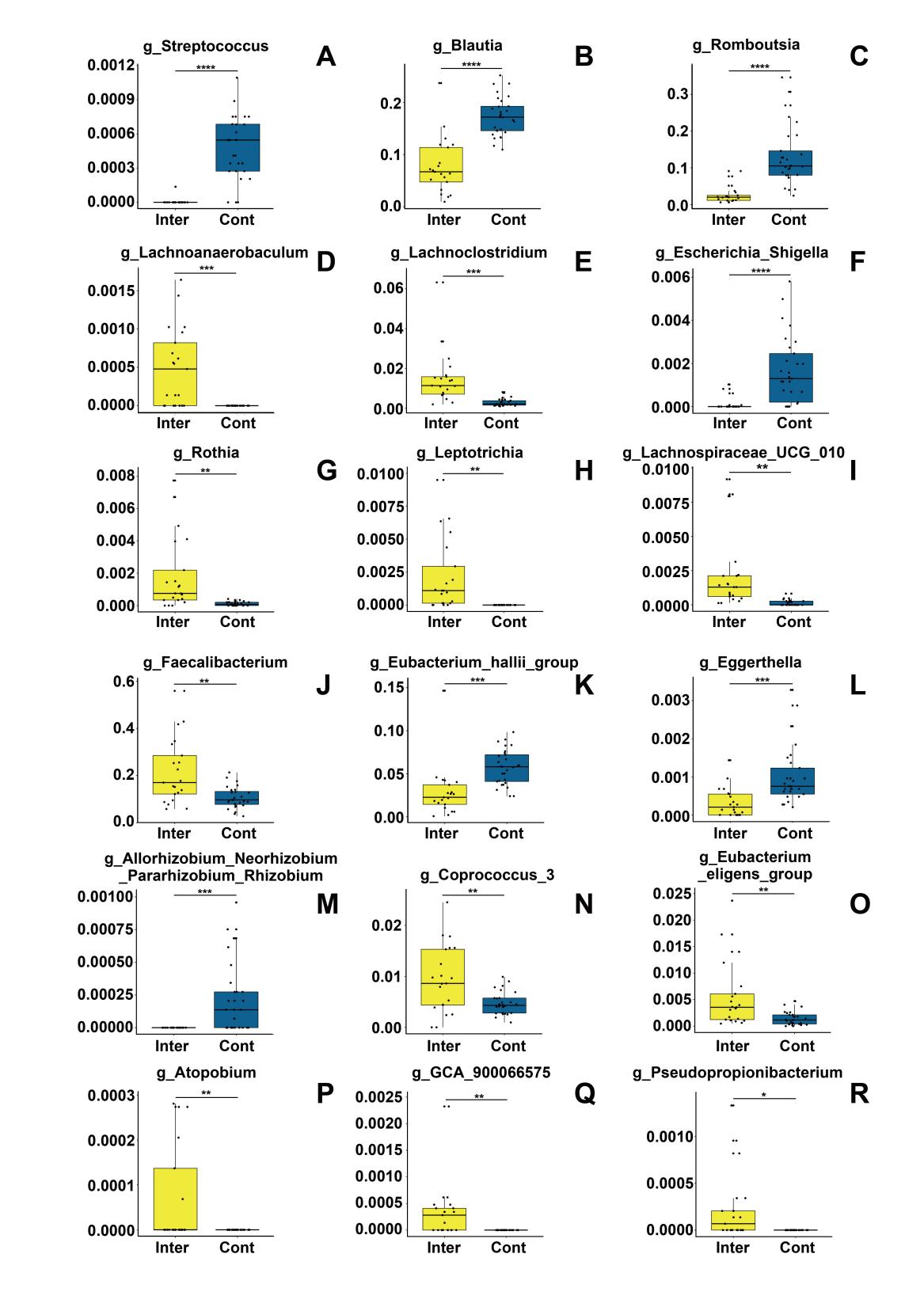


Supplementary Figure 5. Relative abundance of significant genera in different time points among high trait-anxiety group before and after MBCT intervention (at baseline, at the end of intervention, and at the 4-week following the end of intervention): *Streptococcus* (A)*, Blautia* (B)*, Romboutsia* (C)*, Lachnoanaerobaculum* (D)*, Lachnoclostridium* (E), *Escherichia_Shigella* (F)*, Rothia* (G)*, Leptotrichia* (H)*, Lachnospiraceae_UCG_010* (I)*, Faecalibacterium* (J)*, Eubacterium_hallii_group* (K)*, Eggerthella* (L)*, Allorhizobium_Neorhizobium_Pararhizobium_Rhizobium* (M)*, Coprococcus_3* (N)*, Eubacterium_eligens_group* (O)*, Atopobium* (P)*, GCA_900066575* (Q) *and Pseudopropionibacterium* (R)*.*


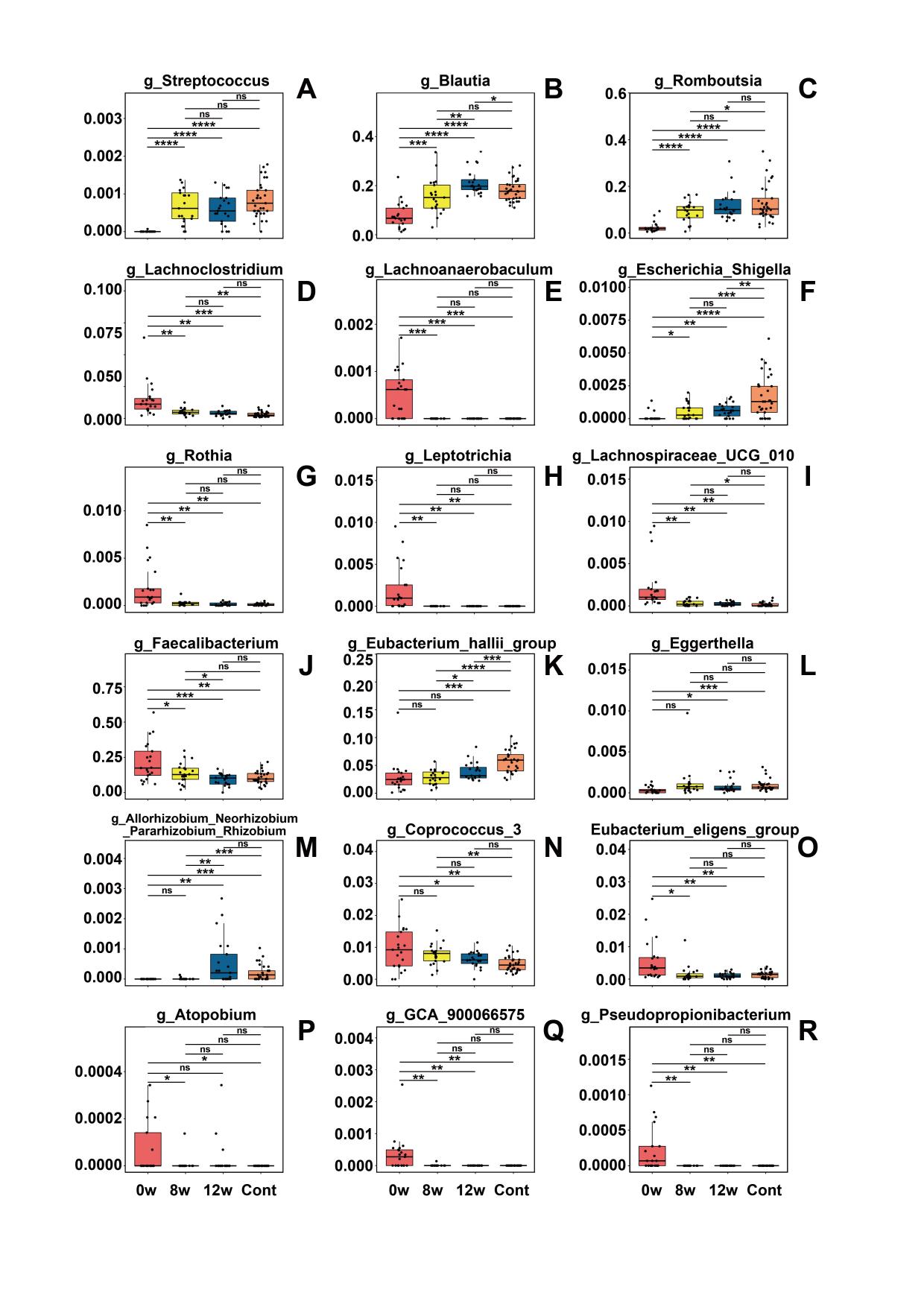


Supplementary figure 6. The differences on alpha diversity between low-responder group and high-responder group before MBCT (0w) (A) and after MBCT (8w) (B).


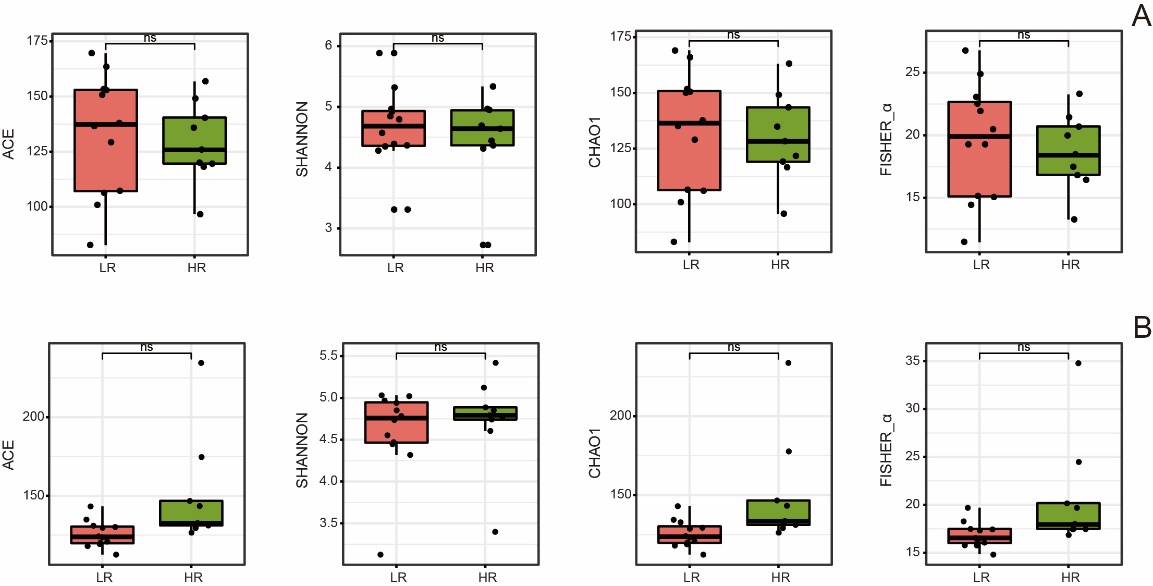


Supplementary Figure 7. Relative abundances at the phylum level, the family level and the genus level of bacterial composition between high-responder group (Sample number in orange color) and low-responder group (Sample number in purple color). (A) Relative abundance at the phylum level. (B) Relative abundance at the family level. (C) Relative abundance at the genus level.


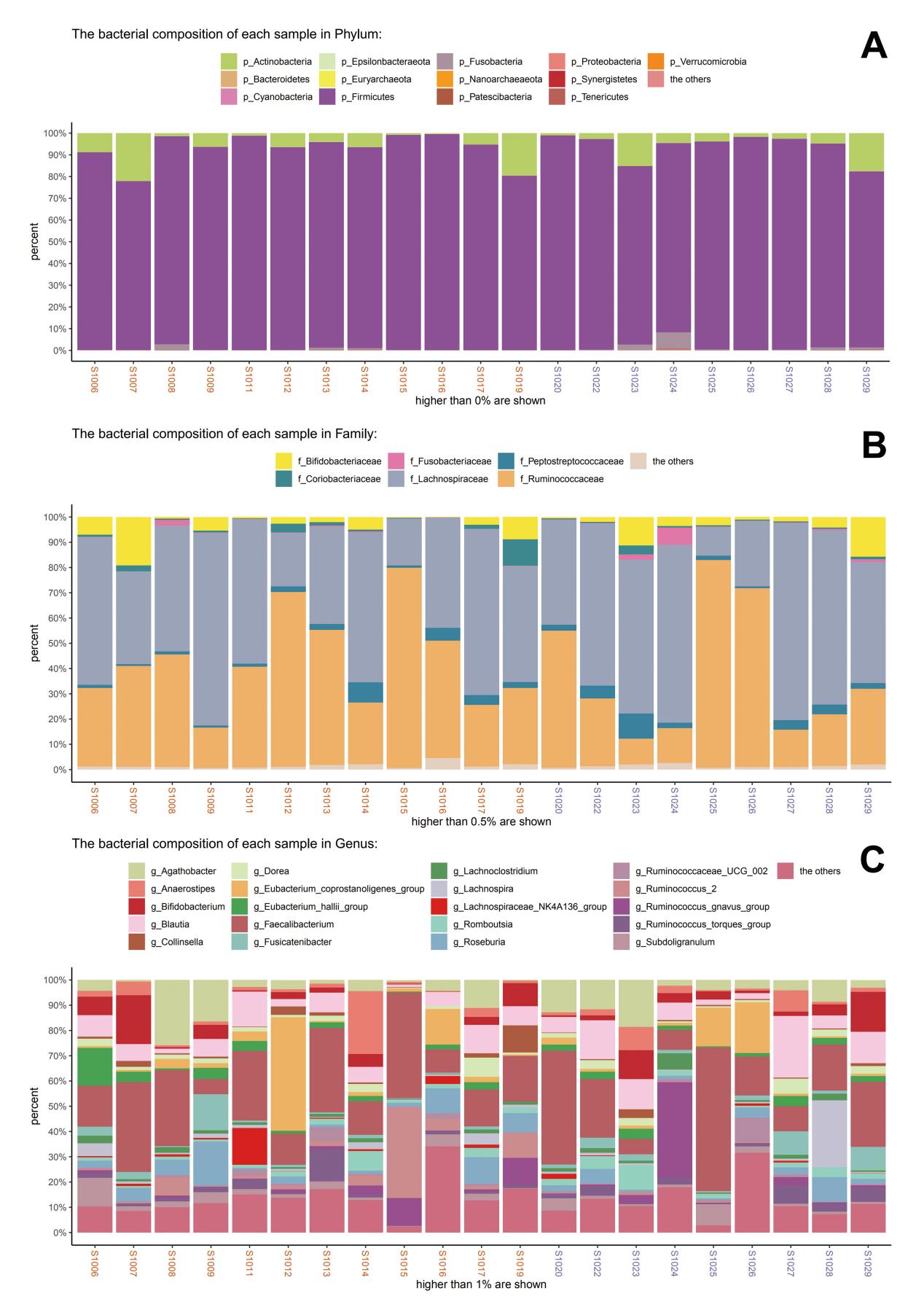


Supplementary figure 8. the alpha and beta diversity differences within high-responder group before MBCT (0w) and after MBCT (8w).


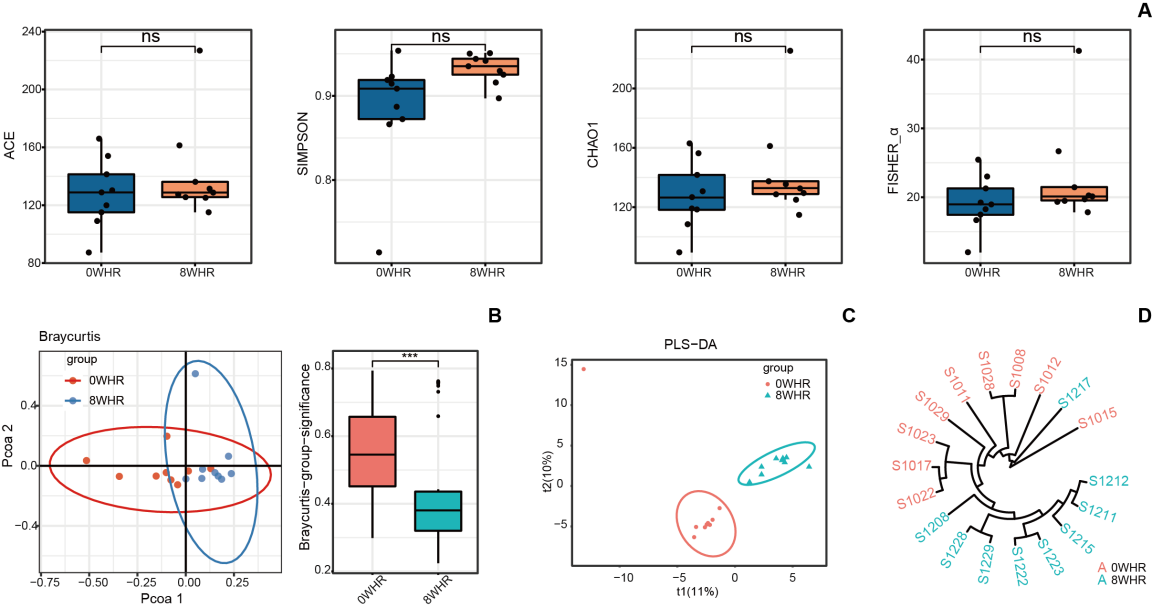


Supplementary Figure 9. The linear correlation between the relative abundance of the genera *Subdoligranulum* levels with psychometric indicators of resilience (A-B), depression (C-D) and mindfulness (E-F) among high-responders (HR) and low-responders (LR).


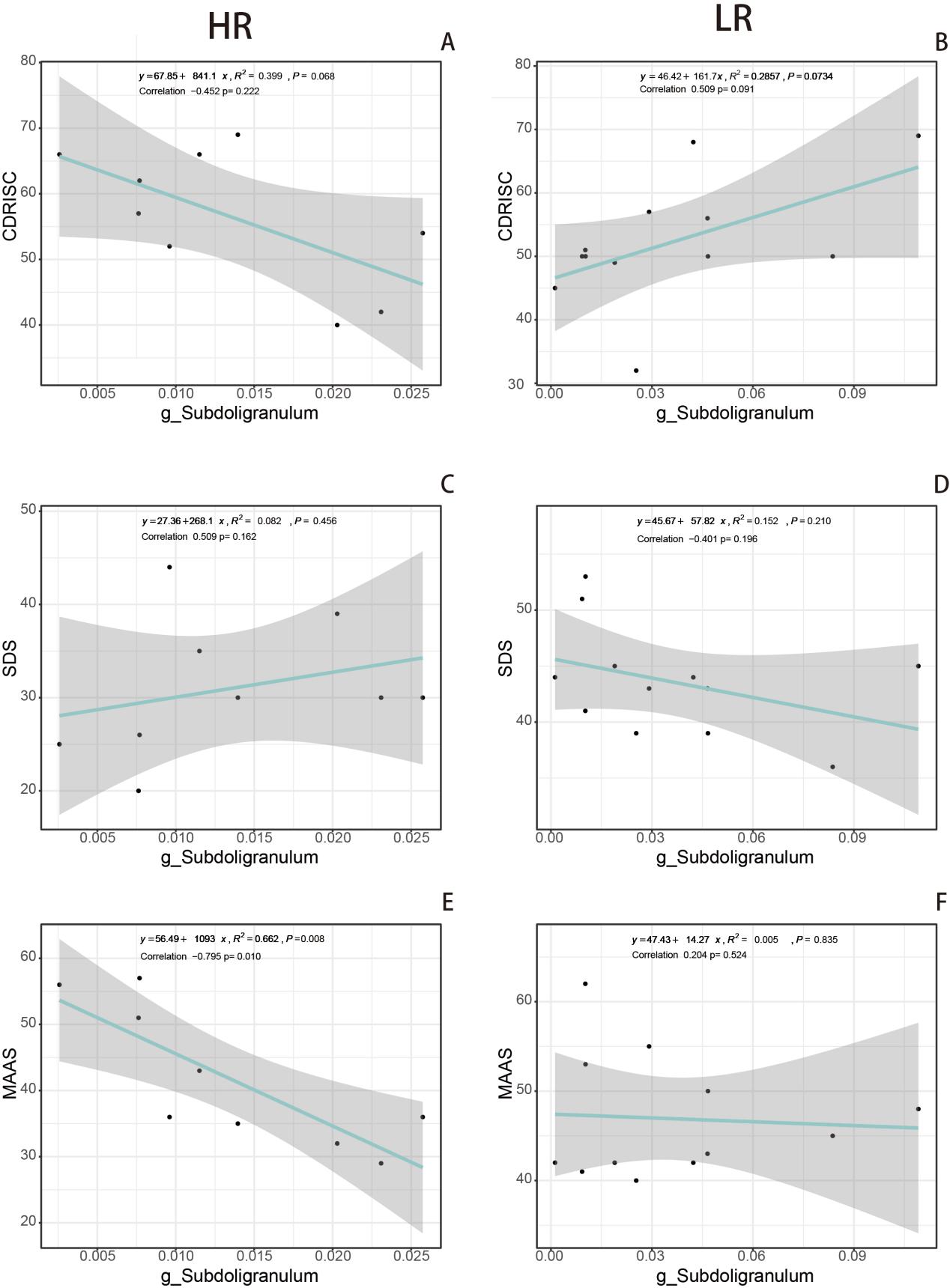

Supplement: Supplementary file 1 [file DataSheet_1.docx]
