# Supplementary material for: Gut Microbiota Associated With Effectiveness And Responsiveness to Mindfulness-Based Cognitive Therapy in Improving Trait Anxiety
Source: Front Cell Infect Microbiol. 2022 Feb 24;12:719829. doi: 10.3389/fcimb.2022.719829 (PMC8908961; doi:10.3389/fcimb.2022.719829)
Supplement: Supplementary file 2 [file Table_1.docx]

**Supplementary Table S1. Mixed model.**

| **Nutrients** | **Mean±SD** | **Coefficients (95%CI)** | **P** |
| --- | --- | --- | --- |
| **Calories (kcal)** |  |  |  |
| 0w | 1625.56±210.24 |  |  |
| 8w | 1088.46±150.88 | -537.10 (-902.72 to -171.47) | 0.006 |
| 12w | 1848.15±216.30 | 222.59 (-300.97 to 746.15) | 0.386 |
| **Protein (g)** |  |  |  |
| 0w | 81.40±14.81 |  |  |
| 8w | 51.44±7.14 | -29.96 (-64.45 to 4.53) | 0.085 |
| 12w | 76.89±7.86 | -4.50 (-40.29 to 31.28) | 0.796 |
| **Fat (g)** |  |  |  |
| 0w | 52.65±7.66 |  |  |
| 8w | 34.27±7.96 | -18.38 (-36.67 to -0.10) | 0.049 |
| 12w | 56.33±6.74 | 3.68 (-15.81 to 23.16) | 0.698 |
| **Fiber (g)** |  |  |  |
| 0w | 6.46±0.92 |  |  |
| 8w | 4.51±1.01 | -1.96 (-4.76 to 0.56) | 0.120 |
| 12w | 5.94±1.03 | -0.53 (-3.02 to 1.96) | 0.663 |
| **Carbohydrate (g)** |  |  |  |
| 0w | 204.64±26.92 |  |  |
| 8w | 143.96±15.63 | -60.69 (-96.89 to -24.48) | 0.002 |
| 12w | 258.10±40.04 | 53.46 (-28.88 to 135.81) | 0.191 |
| **Sugar (g)** |  |  |  |
| 0w | 21.20±5.16 |  |  |
| 8w | 13.34±4.62 | -7.86 (-15.42 to -0.30) | 0.042 |
| 12w | 19.77±6.72 | -1.43 (-18.18 to 15.32) | 0.860 |

Note: Results of mixed models looking at the data longitudinally.
